# Case report: Anti-GAD65 antibody-associated autoimmune encephalitis following HPV vaccination

**DOI:** 10.3389/fneur.2022.1017086

**Published:** 2022-10-05

**Authors:** Aonan Li, Ying Hu, Jialu Li, Xingui Chen, Yubao Jiang, Chengjuan Xie

**Affiliations:** Department of Neurology, The First Affiliated Hospital of Anhui Medical University, Hefei, China

**Keywords:** anti-glutamic acid decarboxylase 65 (anti-GAD65) antibody, autoimmune encephalitis (AE), seizure, human papillomavirus, vaccination, cervical cancer

## Abstract

Human papillomavirus (HPV) infection is a sexually transmitted disease that may lead to cervical cancer. HPV vaccines have been implemented widely to prevent this. While generally few complications of vaccination are reported, there have been occasional reports of adverse reactions post-vaccination. The safety profile of the HPV vaccine is reassuring. However, since its introduction, several serious post-vaccination central nervous system complications have been reported; however, causality has not been established. Herein, we describe a 39-year-old woman who developed seizures and experienced a rapid decline in memory shortly after her first dose of the HPV vaccine. Cranial magnetic resonance imaging and cerebrospinal fluid analysis were performed, and the patient was diagnosed with anti-glutamic acid decarboxylase 65 (anti-GAD65) antibody-associated autoimmune encephalitis. She responded well to high-dose glucocorticoids. Four-month follow-up revealed full recovery and absence of recurrence. Since the HPV vaccine is administered worldwide, this case should raise clinicians' awareness regarding the possible CNS complications related to vaccinations, such as anti-GAD65 antibody-associated AE.

## Introduction

Cervical cancer is one of the most dangerous malignancies affecting women worldwide. An estimated 604,000 new cases of cervical cancer and 342,000 deaths are reported annually (1). Its occurrence is closely related to persistent infection with high-risk human papilloma virus (HPV). Currently, the HPV vaccine is the most important primary measure to prevent cervical cancer. Various HPV vaccinations currently on the market are safe and effective, with the bivalent HPV vaccine being more than 90% effective against HPV 16/18-related precancerous lesions (2, 3). However, adverse reactions to vaccines require equal attention. Several serious neurological disorders following vaccines have been reported, including autoimmune encephalitis (AE), myelitis and central nervous system (CNS) demyelination thus far (4–6).

Autoimmune encephalitis (AE) is a type of encephalitis mediated by mechanisms that induce an immune response against central nervous system antigens. Different subtypes of AEs are distinguished according to antibodies and have different clinical symptoms and prognoses. Among them, AE associated with anti-glutamate decarboxylase-rich antibodies is a treatable cause of encephalitis. Anti-GAD65 antibody-associated AE is characterized by acute or subacute seizures, psychiatric symptoms, and cognitive impairment. Other manifestations include cerebellar ataxia and stiff person syndrome (7–9). The patient may present with a single symptom or multiple symptoms together. In the absence of a detectable paraneoplastic etiology, it is also regarded as a type of limbic encephalitis. It responds well to immunotherapy, including intravenous human immunoglobulin, glucocorticoids, plasma exchange, and other immunosuppressive agents.

In this case report, we describe a 39-year-old woman with anti-GAD65 antibody-associated AE who had seizures and short-term memory deficits shortly after receiving her first dose of HPV vaccination.

## Case description

A healthy 39-year-old female patient suffered a sudden generalized tonic-clonic seizure during sleep 12 days after receiving the first dose of bivalent HPV vaccine (Cecolin^®^, Xiamen Innovax, Xiamen, China). The patient complained of tinnitus, auditory hypersensitivity, dizziness, and memory loss, all of which began after the first seizure. Additionally, the patient complained of difficulty falling asleep, poor continuity of sleep at night, and feeling tired and weak after waking up. There are also symptoms of dysautonomia, such as excessive sweating.

Fever, psychiatric symptoms, or involuntary movements were not observed. The patient did not have any previous physical or mental illnesses, was not under any medication, and had no family history of genetic disorders. There were no abnormalities noted during the pre-HPV vaccination screening for cervical cancer, indicating that the patient did not have an HPV infection prior to vaccination.

**Figure 1:**
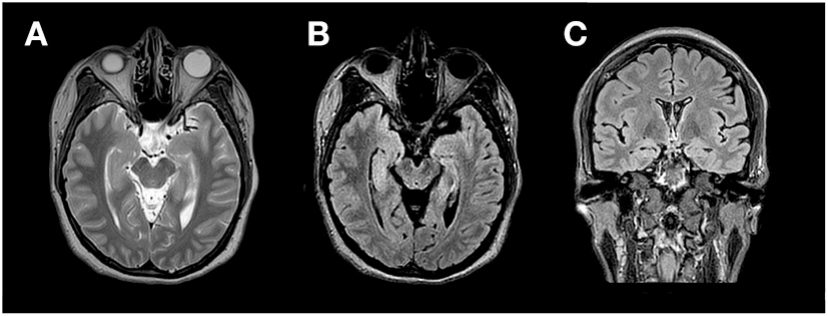
MRI (3.0T) at the time of presentation. Axial T2 Weighted Image **(A)**, Axial Fluid attenuated inversion recovery (FLAIR) sequence **(B)** and coronal FLAIR **(C)** show cortical thickening and hyper intense signal in the medial aspect of bilateral temporal lobes Right > Left.

After her third seizure, the patient was hospitalized for further examination and treatment. She was prescribed oral levetiracetam (1.0 g/day) to control seizures. Neurological examination was normal except for short-term memory impairment. Further assessment of the patient's overall cognitive and memory functions revealed a score of 24/30 (normal range 26–30) on the Montreal Cognitive Assessment (MoCA), showing impaired orientation, attention, and short-term memory. Results of the Auditory Vocabulary Learning Test showed a score of 6 for immediate recall, 9 for delayed recall, and 13 for recognition, indicating impaired memory function. Laboratory tests showed that blood cell count, blood biochemistry, thyroid function, rheumatic disease screening were within the normal range. Tumor marker detection revealed an elevated CA125 level (34.79 U/mL, normal range 0–30.2 U/mL), while the levels of other tumor markers were normal. Semi-quantitative detection of paraneoplastic neuron antibody showed that the GAD65 antibody was positive, with a titer of 32 AU; other antibodies were negative. Thoraco-abdomino-pelvic computed tomography showed a few foci of fibrosis in the middle lobe of the right lung and ovarian cysts on the left side, without other abnormal findings. Pelvic ultrasound and further examination of the bilateral adnexal showed no abnormalities. Subsequently, the findings of pelvic computed tomography were considered a menstrual-related ovarian cyst, and teratoma was excluded. Ultrasound of the thyroid, breast, bilateral neck, and axillary lymph nodes showed a benign right breast nodule and a left thyroid cyst, with no change in size or morphology compared with that of the patient's physical examination conducted 2 years ago. Magnetic resonance imaging of the brain showed abnormal signals in both medial temporal lobes, predominantly on the right side (Figure 1). Video electroencephalogram monitoring revealed widespread diffuse slow waves during wakefulness.

**Figure 2:**
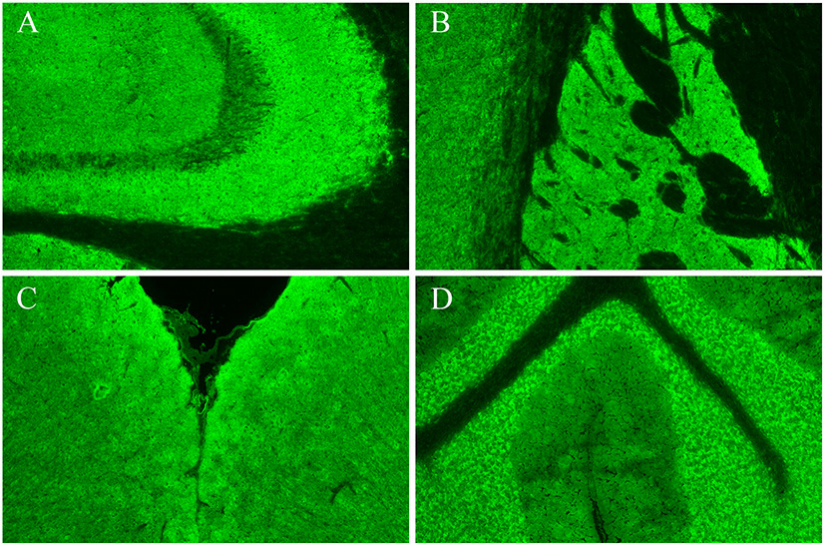
Results of cerebrospinal fluid using tissue-based assays (TBA). Hippocampus **(A)**, striatum **(B)**, cerebral cortex **(C)**, and cerebellum **(D)**.

Cerebrospinal fluid (CSF) analysis showed elevated leukocytes (35 leukocytes, 100% mononuclear cells), normal protein, glucose, and chloride, and no CSF infection. Cell-based and tissue-based assays revealed that the CSF was positive for GAD65 antibody (titer, 1:100++), mainly distributed in the hippocampus, striatum, cerebral cortex, and cerebellum (Figure 2). All other AE-associated antibodies tested negative.

Based on these results, the patient was diagnosed with anti-GAD65 antibody-associated AE. Pulse dose steroid therapy was initiated—high dose glucocorticoids (methylprednisolone, 500 mg daily for 5 days) initially and tapered to 250 mg daily for 5 days, with a good response. Oral prednisone treatment was continued after discharge, initially 60 mg per day then gradually tapered to 5 mg for maintenance. On follow-up, the patient reported no further seizures after discharge, and her memory and sleep quality have improved. After 4 months, the Video EEG retest showed normal results. The MoCA score improved to 30/30, representing an improvement in her overall cognitive function. The immediate recall, delayed recall, and recognition scores in the Auditory Vocabulary Learning Test increased to 10, 13, and 15, respectively, indicating improved memory. Nonetheless, she complained of a persistent subtle decline in memory relative to her premorbid state.

## Discussion

In this report, we describe a woman with generalized tonic-clonic seizure during sleep and short-term memory impairment 12 days after her first dose of the bivalent HPV vaccine. After excluding other causes, the patient's clinical presentation, positive CSF GAD65 antibody, neuroimaging, and video electroencephalogram monitoring findings, led to the diagnosis of anti-GAD65 antibody-associated AE, which meets Graus' criteria for autoimmune encephalitis (10). In patients with AE with seizures as the first symptom, the differential diagnosis should include diseases such as viral encephalitis, metabolic encephalopathy, central nervous system demyelination, etc.

The HPV vaccine is essential for reducing cervical cancer incidence and mortality. Symptoms such as headaches, dizziness, nausea, and muscle pain are common and self-limiting adverse reactions after the HPV vaccination (11, 12). Although the safety and efficacy of HPV vaccines have been demonstrated in clinical trials (13), post-marketing surveillance has turned up multiple reports of serious neurological adverse effects (e.g., such as acute disseminated encephalomyelitis, Guillain-Barré syndrome, multiple sclerosis, optic neuritis, and encephalitis) following vaccination (4, 6, 14–17). Therefore, based on previous reports of CNS adverse reactions following HPV vaccination, clinicians were quick to consider HPV vaccination as a possible trigger for anti-GAD65 antibody-associated AE.

In a study of 1,396 cases of encephalitis after vaccination (hepatitis B, influenza, Haemophilus influenza type B, and measles-mumps-rubella), the onset of encephalitis was within 2 weeks of vaccination in 708 patients (18). This patient had symptoms ~12 days after her vaccination; she had no illness such as fever or respiratory infections before the onset of the disease. Hence, based on the compatible temporal relationship between the onset of symptoms and the presumed trigger, physical examination and diagnostic tests, and the exclusion of an HPV infection through cervical cancer virus screening before vaccination. This patient was diagnosed with anti-GAD65 antibody-associated AE, the first case of autoimmune encephalitis linked to the timing of HPV vaccination.

The association between HPV vaccination and autoimmune encephalitis relates to various factors, including genetic susceptibility, immune dysfunction after viral infection, and the type of vaccine adjuvant. Susceptibility to infection, inflammation, and autoimmune responses vary among individuals, and individual heterogeneity in the immune response has a significant impact on the response after vaccination.

The pathophysiological mechanisms that trigger neuroinflammation after vaccination need to be further investigated. Strong expression of pro-inflammatory cytokines and T-cell responses may be responsible for the neuroinflammation triggered after vaccination. The ChAdOx1 nCoV-19 vaccine has demonstrated this in clinical trials (19). Many target genes are induced and transcribed, leading to the synthesis and release of pyrogenic cytokines into the circulation (12), imitating the body's reaction to a natural infection. Following stimulation, the body produces a complicated set of immune cascade responses. These cytokines and inflammatory mediators in the blood can induce a strong immune response in the nervous system. In some, microglia activation leads to the development of neuroinflammation (12, 20). In addition, there are hypotheses that the link between vaccination and encephalitis may be due to the opening of the blood-brain barrier and disruption of immune tolerance caused by CNS infection, or through molecular mimicry, such as a shared pathogenic epitope between the vaccine (antigen) and CNS structures (21). Nevertheless, this may be the explanation for the possible association between vaccination and autoimmune encephalitis.

Investigations on adverse reactions to vaccines have found that aluminum adjuvants used in vaccines can also trigger autoimmune reactions through specific molecular patterns and non-specific mechanism (22). In addition, aluminum adjuvants can also trigger an acute encephalopathic state by enhancing the immune response to antigens (23). And aluminum adjuvants are also themselves antigens; they can pass through the blood-brain barrier and deposit in the brain, generating neurotoxic effects and compromising cognitive function. Genetic susceptibility matters to the immune response following vaccination. Human leukocyte antigen and non-human leukocyte antigen genes drive the differences in immune responses following vaccination. Previous studies demonstrated an association between different human leukocyte antigen genes and hyper- or hyporesponsiveness to vaccination (24). Therefore, the development of autoimmune-related diseases is more likely among patients with a genetic predisposition to an enhanced response to vaccination (25). However, additional mechanisms may be implicated.

Large-scale and long-term safety data shows that HPV vaccination has not led to an increased incidence of autoimmune disease (26–28). It is possible that no direct association between vaccination and disease exists in this patient. Post-vaccination, some cases of neurological disease may arise only by chance. Because this is a case report, we admit that a causal relationship between vaccination and encephalitis cannot be proven. Observational data or animal models will be needed to determine causality. Moreover, we continue to believe that the advantages of vaccination significantly outweigh the possible hazards of an ongoing immunization program.

## Conclusion

We report the first case of anti-GAD65 antibody-associated AE following HPV vaccination in a patient who recovered well without severe cognitive impairment due to prompt diagnosis and treatment. However, the etiology of autoimmune encephalitis after HPV vaccination is not completely clear and several possible mechanisms may be involved and must be further investigated. For now, it is important to collect reports of autoimmune encephalitis after vaccination. Therefore, this case report provides clinicians with the opportunity to identify potentially vaccine-associated anti-GAD65 antibody-associated AE. In the meantime, clinicians should be vigilant in inquiring about the vaccination history of patients with AE and may need to pay particular attention to patients with anti-GAD65 antibody-associated AE who were injected with the HPV vaccine.

## Patient perspective

When the patient had her first seizure, she and her family were very worried. Thorough investigation post admission revealed the actual cause of seizure, which brought relief to the patient and her family. After several days of glucocorticoid pulse therapy, seizure recurrence ceased, and her memory gradually recovered. The patient learned that her encephalitis was most likely a rare adverse reaction related to vaccination and was happy to share her case. At the same time, she hopes that people with the same condition will be as fortunate to be diagnosed and treated promptly.

## Data availability statement

The original contributions presented in the study are included in the article/Supplementary material, further inquiries can be directed to the corresponding author/s.

## Ethics statement

The studies involving human participants were reviewed and approved by Ethics Committee of Anhui Medical University. The patients/participants provided their written informed consent to participate in this study. Written informed consent was obtained from the individual(s) for the publication of any potentially identifiable images or data included in this article.

## Author contributions

AL compiled background information and wrote the manuscript. YH, JL, and XC acquired and analyzed data. YH, YJ, and XC were treating physicians. AL and CX revised the manuscript. All authors approved the final manuscript.

## Funding

This study was supported by the Anhui Provincial Natural Science Foundation (No. 1908085MH249).

## Conflict of interest

The authors declare that the research was conducted in the absence of any commercial or financial relationships that could be construed as a potential conflict of interest.

## Publisher's note

All claims expressed in this article are solely those of the authors and do not necessarily represent those of their affiliated organizations, or those of the publisher, the editors and the reviewers. Any product that may be evaluated in this article, or claim that may be made by its manufacturer, is not guaranteed or endorsed by the publisher.

## Abbreviations

AE: autoimmune encephalitis
anti-GAD65: anti-glutamic acid decarboxylase 65
CSF: cerebrospinal fluid
GABA: gamma-aminobutyric acid
HPV: human papilloma virus.

## References

[1] Sung H Ferlay J Siegel RL Laversanne M Soerjomataram I Jemal A. Global cancer statistics 2020: GLOBOCAN estimates of incidence and mortality worldwide for 36 cancers in 185 countries. CA Cancer J Clin. (2021) 71:209–49. 10.3322/caac.21660 33538338

[2] Centers for Disease Control and Prevention (CDC). FDA licensure of bivalent human papillomavirus vaccine (HPV2, Cervarix) for use in females and updated HPV vaccination recommendations from the Advisory Committee on Immunization Practices (ACIP). MMWR Morb Mortal Wkly Rep. (2010) 59:626–9. 20508593

[3] Paavonen J Naud P Salmerón J Wheeler CM Chow SN Apter D. Efficacy of human papillomavirus (HPV)-16/18 AS04-adjuvanted vaccine against cervical infection and precancer caused by oncogenic HPV types (PATRICIA): final analysis of a double-blind, randomised study in young women. Lancet. (2009) 374:301–14. 10.1016/S0140-6736(09)61248-4 19586656

[4] Hviid A Svanstrom H Scheller NM Gronlund O Pasternak B Arnheim-Dahlstrom L. Human papillomavirus vaccination of adult women and risk of autoimmune and neurological diseases. J Intern Med. (2018) 283:154–65. 10.1111/joim.12694 29044769

[5] Martin S Azzouz B Morel A Trenque T. Anti-NMDA receptor encephalitis and vaccination: a disproportionality analysis. Front Pharmacol. (2022) 13:940780. 10.3389/fphar.2022.940780 36059934 PMC9428621

[6] Sekiguchi K Yasui N Kowa H Kanda F Toda T. Two cases of acute disseminated encephalomyelitis following vaccination against human papilloma virus. Intern Med. (2016) 55:3181–4. 10.2169/internalmedicine.55.5472 27803416 PMC5140871

[7] Graus F Saiz A Dalmau J. GAD antibodies in neurological disorders - insights and challenges. Nat Rev Neurol. (2020) 16:353–65. 10.1038/s41582-020-0359-x 32457440

[8] Honnorat J Plazat LO. Autoimmune encephalitis and psychiatric disorders. Rev Neurol. (2018) 174:228–36. 10.1016/j.neurol.2017.11.004 29609960

[9] Vrillon A Carle G Berzero G Honnorat J Huberfeld G Psimaras D. Psychiatric symptoms in anti glutamic acid decarboxylase associated limbic encephalitis in adults: a systematic review. Neurosci Biobehav Rev. (2020) 119:128–37. 10.1016/j.neubiorev.2020.08.015 33022299

[10] Graus F Titulaer MJ Balu R Benseler S Bien CG Cellucci T. A clinical approach to diagnosis of autoimmune encephalitis. Lancet Neurol. (2016) 15:391–404. 10.1016/S1474-4422(15)00401-9 26906964 PMC5066574

[11] Bonaldo G Vaccheri A D'Annibali O Motola D. Safety profile of human papilloma virus vaccines: an analysis of the US Vaccine Adverse Event Reporting System from 2007 to 2017. Br J Clin Pharmacol. (2019) 85:634–43. 10.1111/bcp.13841 30569481 PMC6379209

[12] Herve C Laupeze B Del Giudice G Didierlaurent AM Tavares Da Silva F. The how's and what's of vaccine reactogenicity. NPJ Vaccines. (2019) 4:39. 10.1038/s41541-019-0132-6 31583123 PMC6760227

[13] Wheeler CM Castellsagué X Garland SM Szarewski A Paavonen J Naud P. Cross-protective efficacy of HPV-16/18 AS04-adjuvanted vaccine against cervical infection and precancer caused by non-vaccine oncogenic HPV types: 4-year end-of-study analysis of the randomised, double-blind PATRICIA trial. Lancet Oncol. (2012) 13:100–10. 10.1016/S1470-2045(11)70287-X 22075170

[14] Grimaldi-Bensouda L Rossignol M Kone-Paut I Krivitzky A Lebrun-Frenay C Clet J. Risk of autoimmune diseases and human papilloma virus (HPV) vaccines: Six years of case-referent surveillance. J Autoimmun. (2017) 79:84–90. 10.1016/j.jaut.2017.01.005 28190705

[15] Mouchet J Salvo F Raschi E Poluzzi E Antonazzo IC De Ponti F. Human papillomavirus vaccine and demyelinating diseases-A systematic review and meta-analysis. Pharmacol Res. (2018) 132:108–18. 10.1016/j.phrs.2018.04.007 29665426

[16] Martinez-Lavin M. HPV vaccine: adverse event signals were minimised or ignored. BMJ. (2019) 366:l4508. 10.1136/bmj.l4508 31278075

[17] Anamnart C Tisavipat N Owattanapanich W Apiwattanakul M Savangned P Prayoonwiwat N. Newly diagnosed neuromyelitis optica spectrum disorders following vaccination: case report and systematic review. Mult Scler Relat Disord. (2022) 58:103414. 10.1016/j.msard.2021.103414 35216789

[18] Al Qudah Z Abukwaik W Patel H Souayah N. Encephalitis after Vaccination in United States. A Report from the CDC/FDA Vaccine Adverse Event Reporting System. [1990–2010]. Neurology. (2012) 78:P03.151. 10.1212/WNL.78.1_MeetingAbstracts.P03.151

[19] Ewer KJ Barrett JR Belij-Rammerstorfer S Sharpe H Makinson R Morter R. T cell and antibody responses induced by a single dose of ChAdOx1 nCoV-19 (AZD1222) vaccine in a phase 1/2 clinical trial. Nat Med. (2021) 27:270–8. 10.1038/s41591-020-01194-5 33335323

[20] Giannotta G Giannotta N. Vaccines and neuroinflammation. Int J Pub Heal Safe. (2018) 3:1000163.

[21] Huynh W Cordato DJ Kehdi E Masters LT Dedousis C. Post-vaccination encephalomyelitis: literature review and illustrative case. J Clin Neurosci. (2008) 15:1315–22. 10.1016/j.jocn.2008.05.002 18976924 PMC7125578

[22] Salemi S D'Amelio R. Could autoimmunity be induced by vaccination? Int Rev Immunol. (2010) 29:247–69. 10.3109/08830181003746304 20521925

[23] Khan Z Combadière C Authier FJ Itier V Lux F Exley C. Slow CCL2-dependent translocation of biopersistent particles from muscle to brain. BMC Med. (2013) 11:99. 10.1186/1741-7015-11-99 23557144 PMC3616851

[24] Ovsyannikova IG Poland GA. Vaccinomics: current findings, challenges and novel approaches for vaccine development. AAPS J. (2011) 13:438–44. 10.1208/s12248-011-9281-x 21671143 PMC3160164

[25] Posteraro B Pastorino R Di Giannantonio P Ianuale C Amore R Ricciardi W. The link between genetic variation and variability in vaccine responses: systematic review and meta-analyses. Vaccine. (2014) 32:1661–9. 10.1016/j.vaccine.2014.01.057 24513009

[26] Willame C Rosillon D Zima J Angelo MG Stuurman AL Vroling H. Risk of new onset autoimmune disease in 9- to 25-year-old women exposed to human papillomavirus-16/18 AS04-adjuvanted vaccine in the United Kingdom. Hum Vaccin Immunother. (2016) 12:2862–71. 10.1080/21645515.2016.1199308 27428517 PMC5137515

[27] Lehtinen M Eriksson T Apter D Hokkanen M Natunen K Paavonen J. Safety of the human papillomavirus (HPV)-16/18 AS04-adjuvanted vaccine in adolescents aged 12–15 years: Interim analysis of a large community-randomized controlled trial. Hum Vaccin Immunother. (2016) 12:3177–85. 10.1080/21645515.2016.1183847 27841725 PMC5215585

[28] Phillips A Hickie M Totterdell J Brotherton J Dey A Hill R. Adverse events following HPV vaccination: 11 years of surveillance in Australia. Vaccine. (2020) 38:6038–46. 10.1016/j.vaccine.2020.06.039 32709432

