# Supplementary material for: Case report: Anti-GAD65 antibody-associated autoimmune encephalitis following HPV vaccination
Source: Front Neurol. 2022 Oct 5;13:1017086. doi: 10.3389/fneur.2022.1017086 (PMC9579373; doi:10.3389/fneur.2022.1017086)
Supplement: Supplementary file 1 [file Data_Sheet_1.docx]

Supplementary Material

# Supplementary Tables

Laboratory Investigations in Blood and Cerebrospinal Fluid (CSF) Done in Patient

| Blood investigation | Standardized investigation of CSF | Tumor marker and Semi-quantitative detection of paraneoplastic neuron antibody in serum | Autoimmune encephalitis antibody investigation in CSF |
| --- | --- | --- | --- |
| Cell count, kidney and liver function, thyroid function, Electrolytes, HIV, Syphilis blood test, Rheumatic Disease Screening (ESR, ASO, CRP, ANA, RF, Ena‐Screen, ANCA‐Screen, anti‐ds DNA, C3c, C4, IgG, IgA, IgM) | CSF aspect, White blood cell count (10^6^/L), Red blood cell count (10^6^/L), Monocytes (%), Total Proteins (g/L), Glucose (mmol/L), Chlorine (mmol/L), Bacteria, Mould, Cryptococci, Acid-fast bacilli, Rubella IgG/IgM, Herpes Simplex Virus IgG/IgM, Cytomegaly Virus IgG/IgM, Toxoplasma IgG/IgM, Epstein Barr Virus IgG/IgM | CEA, AFP, CA125, CA19-9, CA15-3, β-HCG, NSE, HE4, SCC, GH, SF, CA72-4, CYFRA21-1  Hu, Yo, Ri, CV2/CRMP5, Ma1, Ma2, Amphiphysin, SOX1, DNER/Tr, Zic4 GAD65, PKCγ, Recoverin, Titin antibodies | GAD65, NMDA‐, and GABA‐B‐receptor, IgLON5, AMPA‐R subtype 1/2, DPPX, LGI1, CASPR2, Glycine‐receptor, mGluR5, D2R |

# Supplementary Figures


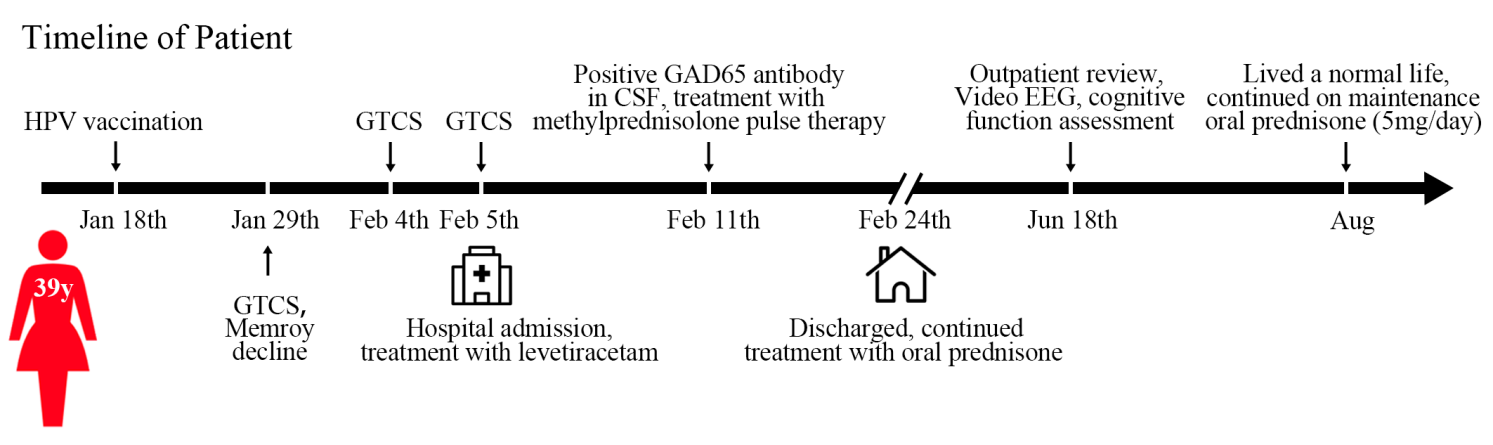


## Supplementary Figures 1. Timeline of patient with relevant data of the episodes and treatments.


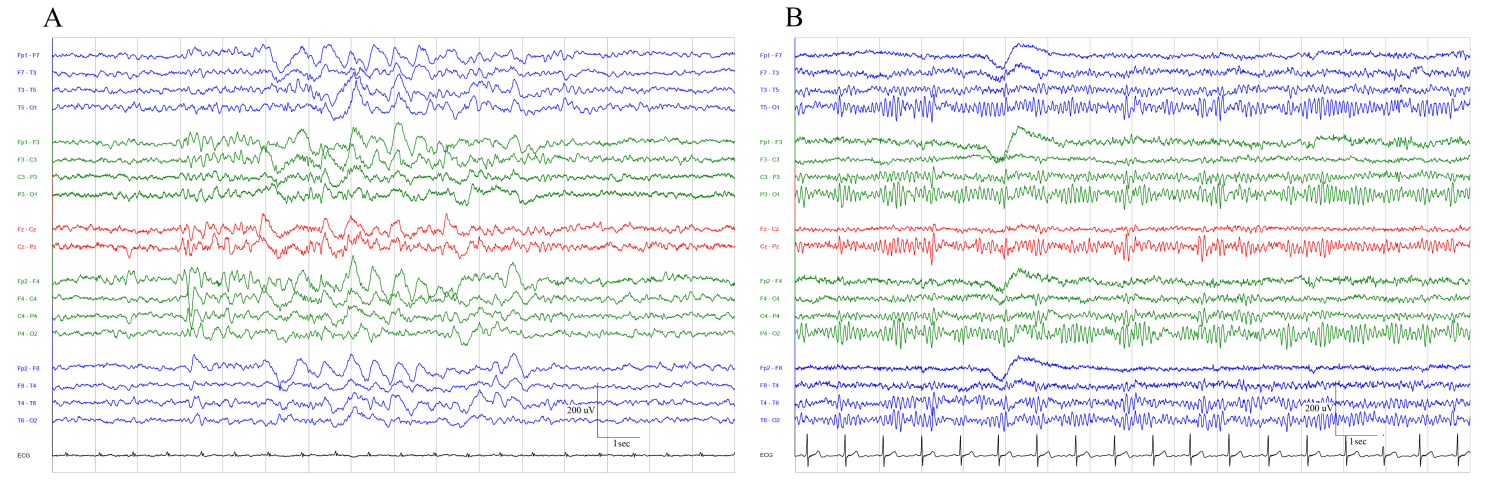
**Supplementary Figures 2.** The first video EEG showed bilateral predominantly short bursts of high potential 2 - 3 c/s slow wave activity in the anterior head interspersed with a single sharp wave emission during the waking period (A). The follow-up EEG after 4 months showed that all stages were normal (B). Representative electroencephalographic recordings from patient using A - P (anterior - posterior) longitudinal bipolar montage (sensitivity 10 mcV/mm; filter 0.5 to 50 Hz; interval between 2 vertical lines: 1 seconds).

**
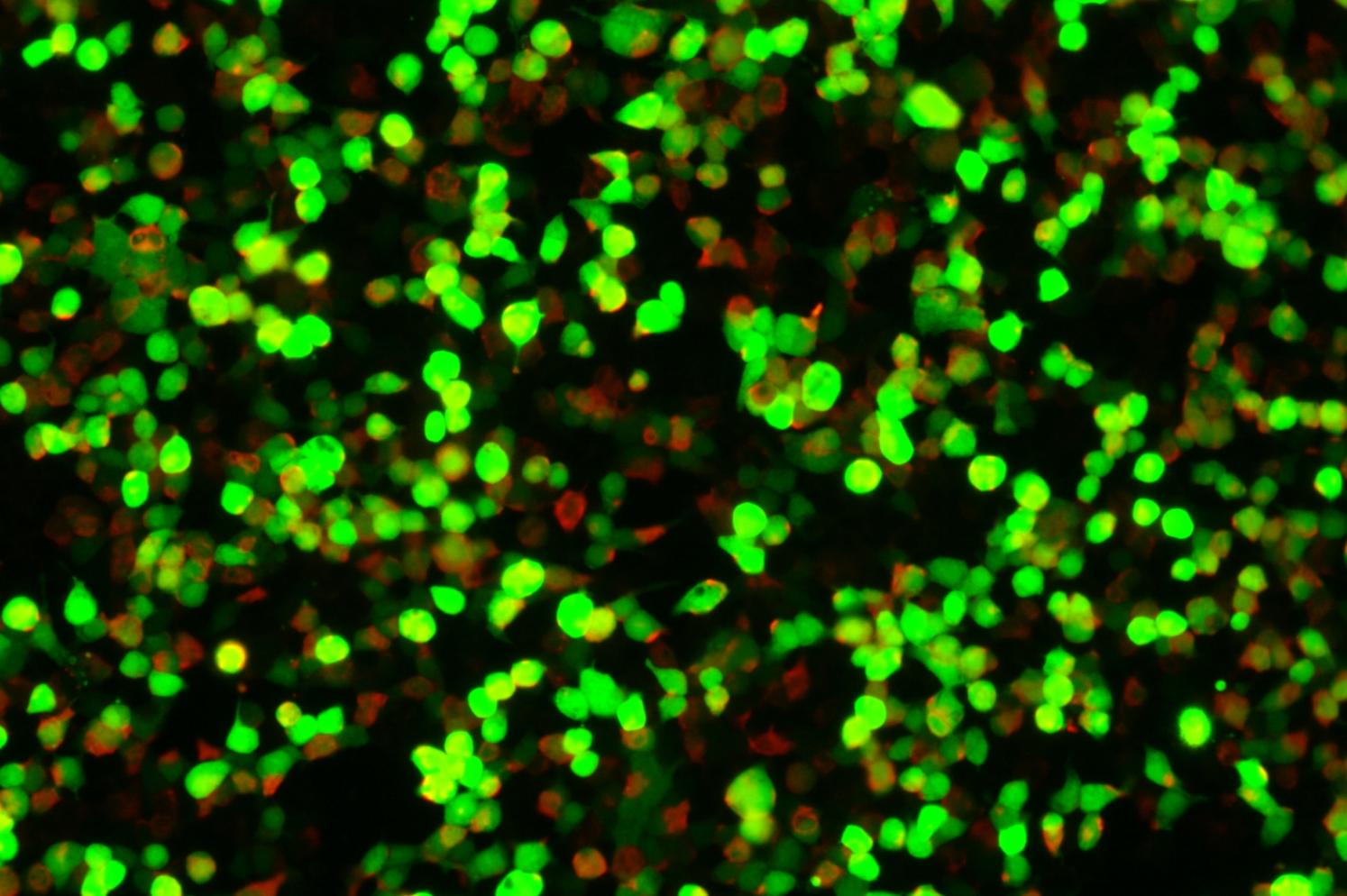
**

**Supplementary Figures 3.** Immunofluorescence assay graph. Before Patient received treatment, the autoimmune encephalitis antibody test was performed and showed positive GAD65 antibody in CSF (The titer was 1:100++).
